# Clarification

**Published:** 2013-01-25

## Vol. 61, No. 48

In the *MMWR* report, “Chlorine Gas Release Associated with Employee Language Barrier — Arkansas, 2011,” it was incorrectly stated that a Spanish-speaking employee with limited English skills inadvertently poured sodium hypochlorite into a 55-gallon drum containing residual acidic antimicrobial solution, causing a chlorine gas release. The drum was labeled only in English. CDC subsequently has learned that, although the Spanish-speaking employee retrieved the 55-gallon drum for mixing because he thought it contained sodium hypochlorite, the actual dispensing of the sodium hypochlorite into the drum was performed by an English-speaking supervisor. The supervisor told investigators he did not read the label on the drum, which he noted appeared similar to other drums containing sodium hypochlorite.

